# Self-Entrapment of Antimicrobial Peptides in Silica Particles for Stable and Effective Antimicrobial Peptide Delivery System

**DOI:** 10.3390/ijms242216423

**Published:** 2023-11-16

**Authors:** Mi-Ran Ki, Sung Ho Kim, Tae In Park, Seung Pil Pack

**Affiliations:** 1 Department of Biotechnology and Bioinformatics, Korea University, Sejong-ro 2511, Sejong 30019, Republic of Korea; 2 Institute of Industrial Technology, Korea University, Sejong-ro 2511, Sejong 30019, Republic of Korea

**Keywords:** antimicrobial peptide, cell penetrating peptide, silica forming peptide, biomimetic silica deposition, drug delivery, drug device combination

## Abstract

Antimicrobial peptides (AMPs) have emerged as a promising solution to tackle bacterial infections and combat antibiotic resistance. However, their vulnerability to protease degradation and toxicity towards mammalian cells has hindered their clinical application. To overcome these challenges, our study aims to develop a method to enhance the stability and safety of AMPs applicable to effective drug–device combination products. The KR12 antimicrobial peptide was chosen, and in order to further enhance its delivery and efficacy the human immunodeficiency virus TAT protein-derived cell-penetrating peptide (CPP) was fused to form CPP-KR12. A new product, CPP-KR12@Si, was developed by forming silica particles with self-entrapped CPP-KR12 peptide using biomimetic silica precipitability because of its cationic nature. Peptide delivery from CPP-KR12@Si to bacteria and cells was observed at a slightly delivered rate, with improved stability against trypsin treatment and a reduction in cytotoxicity compared to CPP-KR12. Finally, the antimicrobial potential of the CPP-KR12@Si/bone graft substitute (BGS) combination product was demonstrated. CPP-KR12 is coated in the form of submicron-sized particles on the surface of the BGS. Self-entrapped AMP in silica nanoparticles is a safe and effective AMP delivery method that will be useful for developing a drug–device combination product for tissue regeneration.

## 1. Introduction

The implantation of medical devices may be necessary to treat or repair damaged tissue. During transplantation, perioperative systemic antibiotic administration is standard practice to prevent infections associated with the implanted device [1]. However, once an infection occurs, systemic antibiotic treatment is often ineffective [2]. Most implant-related infections are antibiotic-resistant and last until the device is removed [2,3]. One effective approach to preventing device and wound infections is the topical administration of antibacterial agents [2]. A strategy to achieving topical antibiotic delivery in a drug–device combination is to integrate an antibacterial agent with a medical device.

Biofilms and device-related infections are typically complex; therefore, it is advisable to select broad-spectrum antibacterial agents, a requirement satisfied by antimicrobial peptides (AMPs) and chitosan in some research studies [4,5]. AMPs are peptides that have developed in organisms ranging from prokaryotes to humans to give non-specific immune protection [6]. Comprising 10 to 60 amino acid residues, AMPs share two common and functionally vital characteristics: an amphipathic structure composed of cationic residues with a net positive charge (+2 to +9), attracting them to anionic microbial surfaces, and hydrophobic residues that preferentially insert into microbial membranes [7]. AMPs have a broad antimicrobial spectrum [7]. They are less likely to induce resistance because of their physical membrane disruption mechanism, which causes the leakage of microbial contents and contributes to bacterial killing [8]. In contrast, conventional antibiotics face increasing resistance, making AMPs an attractive option for treating bacterial infections [9]. Therefore, AMPs are promising therapeutic agents for medical device combination products [2]. Meanwhile, cell-penetrating peptides (CPPs), which are cationic, have been used as vectors to transport oligonucleotides, proteins, peptides, and other materials across bio-membranes into cells because of their ability to cross cell membranes in a non-disruptive manner [10]. Thus, CPPs have received considerable attention for their potential applications in drug delivery to the hard-to-permeate membranes [11]. The combination of CPPs and AMPs has been explored as a viable approach to treat intracellular infections by providing the dual function of cellular penetration and antimicrobial activity [12,13], demonstrating improved antimicrobial activity over AMPs alone [12,14]. Despite the advantages of CPP-conjugated AMPs, rapid clearance and cytotoxicity, such as hemolysis, characteristic of peptide and protein drugs, limit the development of AMPs as systemic antimicrobial agents [15]. Therefore, strategies are needed to incorporate AMPs into medical devices for local release while protecting the peptide from the environment. In developing antimicrobial device combination products, it is crucial to ensure that the antimicrobial agent remains stable during manufacturing and storage [16,17]. This may require a biocompatible delivery system that can protect the drug and enable its controlled release without affecting the overall performance of the medical device.

Understanding how diatoms biologically form their silica skeleton [18] has led to the development of a biocompatible method for creating silica hybrids by mimicking the positively charged peptides involved in silica deposition [19,20,21]. Silica-precipitable peptides have been employed for various applications, such as enzyme immobilization [22], drug delivery vehicle formation [23], and biosilica coatings on bone graft substitutes (BGSs) [24] using the biomimetic silicification technique.

**Scheme 1:**
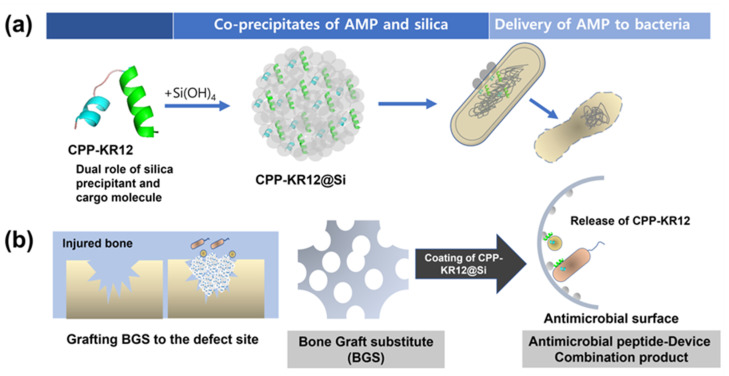
Self-entrapment of AMPs in silica matrix through CPP-KR12-mediated silica deposition (**a**) and antimicrobial peptide-device combination product (**b**).

Our work proposes a new approach to enhance the stability of AMPs, and topical coating of AMPs on medical devices. AMPs and CPPs are expected to possess biomimetic silica precipitability because of their cationic nature. To test this hypothesis, we employed KR12, the shortest human cathelicidin LL37-derived peptide identified as the region responsible for LL37′s antibacterial and anti-inflammatory properties [12,25,26], along with the CPP from the 49th–57th residues (protein transduction domain) of the TAT protein, which is a protein involved in the transcription of the human immunodeficiency virus (HIV) [27]. These AMPs played a dual role as silica-precipitable agents and cargo molecules (Scheme 1a). Furthermore, the feasibility of developing a combination product of AMP-BGS, coated with AMPs in the form of silica particles on the surface of the BGS, was explored (Scheme 1b). A summary table with the nomenclature and a brief description of the formulation/composition of the different systems in this study is shown in Supplementary Table S1.

## 2. Results and Discussion

### 2.1. Conjugation of Cell Penetrating Peptide to KR12 Antimicrobial Peptide

#### 2.1.1. CPP Conjugation Enhances the Antibacterial Activity of KR12 over KR12 Alone

The amino acid sequences and properties of KR12, CPP, and CPP-KR12, in which CPP is bound to KR12 by a GSS linker, are shown in Table 1. The KR12 used in this study is a cationic and amphipathic α-helical peptide composed of the 18–29th residues of LL37, which is a 37-residue, cathelicidin-derived antimicrobial peptide found in humans [25]. In addition, KR12 is known for its ability to promote osteogenic differentiation of bone marrow stem cells [28]. Therefore, it was selected for its potential to serve dual purposes—as an antibacterial agent and to promote bone regeneration—in the production of a combination of antibacterial bone grafts. Cell-penetrating peptides (CPPs) are able to cross biological membranes without membrane disruption; therefore, they have been used as a vector for the delivery of various cargos [29]. The CPP used in this study, from the 49th–57th residues of the TAT protein, have facilitated the cellular uptake of linked bioactive macromolecules such as peptides, proteins, oligonucleotides, and pharmacological compounds [30].

The antimicrobial activity of these peptides was compared by determining the minimum inhibitory concentration (MIC) (Table 2).

KR12 showed antibacterial activity against the Gram-negative bacteria *Escherichia coli* (*E. coli*) and *Pseudomonas aeruginosa* (*P. aeruginosa*), with MIC values in the range of 100–200 μM, indicating low antibacterial potency. It did not exhibit antibacterial activity against the Gram-positive bacteria *Staphylococcus aureus* (*S. aureus*), even at the highest concentration tested (320 μM). CPP did not display antibacterial activity against any bacterial strain, even at the highest concentration tested (320 μM) (Supplementary Figure S1). In contrast, the antibacterial activity of CPP-KR12 increased approximately 8-fold against *E. coli* (*p* = 0.248) and approximately 30-fold against *P. aeruginosa* (*p* = 0.035) Against *S. aureus*, it exhibited a MIC (22.80 ± 8.48 μM) at a concentration 10 times lower than KR12 (*p* = 0), indicating improved antibacterial activity against both Gram-negative and Gram-positive bacteria.

These results were similar to the report by Lee et al., where the conjugation of nine arginine residues to AMP significantly enhanced its antimicrobial activity, especially against Gram-negative bacteria [14]. Although CPP itself did not demonstrate antimicrobial activity, CPP-fused KR12 inhibited cell growth at lower concentrations, suggesting that the cellular internalization ability of CPP enhanced the delivery of KR12 into bacteria. TAT-KR-12 (YGRKKRRQRRKRIVQRIKDFLR), developed for the treatment of intracellular S. aureus infection in mammals, showed enhanced antibacterial activity against *S. aureus* [12]. KR12 alone may be limited in its ability to penetrate thick cell walls and induce cell membrane rupture. However, incorporating cell-penetrating peptides enhances binding affinity to anionic teichoic acid on the cell wall surface because of increased cationicity [31]. This accumulation of AMP on the cell wall surface may ultimately facilitate the insertion of peptides into the cell membrane through the somewhat porous reticulated peptidoglycan layer [32].

#### 2.1.2. Comparison of AMP’s Membrane Permeability and Ability to Disrupt Cell Membranes

The SYTOX^TM^ Green uptake assay was used to evaluate the permeability of the cell membrane induced by the peptides. When AMPs induce membrane permeability, SYTOX^TM^ green dye enters the interior of the cell and binds to intracellular nucleic acids, resulting in increased fluorescence. The disruption of membrane integrity by peptides increases fluorescence intensity, providing an indirect measure of antimicrobial activity as SYTOX^TM^ Green cannot pass through an intact membrane.

**Figure 1:**
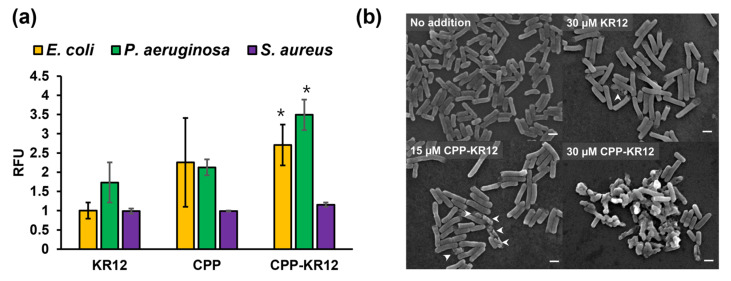
(**a**) SYTOX^TM^ Green uptake assay analyzing membrane permeability in the indicated bacterial cells over KR12, CPP, and CPP-KR12. Relative fluorescence unit (RFU) value represents fluorescence ratio relative to that measured in the absence of peptide in each strain after 10 min exposure of indicated peptide (30 μM). Values are presented as the mean ± SE (N = 3). * *p* < 0.05 vs. KR12 in each strain. (**b**) Scanning electron microscopy (SEM) image of *E. coli* treated with indicated AMP. The image is a 10,000× magnification, and the scale bar represents 1 µm. White arrowheads indicate fragmented cells.

The fluorescence intensity of CPP and CPP-KR12 tended to increase relative to KR12 in Gram-negative bacteria, as shown in Figure 1a. CPP did not show a significant increase relative to KR12, while CPP-KR12 showed a 2-fold increase in fluorescence intensity relative to KR12. However, the Gram-positive bacterium *S. aureus* did not show a difference in uptake among the peptides.

Next, upon investigating the killing mechanism, we utilized scanning electron microscopy (SEM) to observe the morphology of *E. coli* cells after treatment with AMPs (Figure 1b). The results showed that fragmented cells or cells with surface holes were observed, while the majority remained intact in both 30 μM KR12- and 15 μM CPP-KR12-treated *E. coli*. We noted an increase in fragmented cells at 15 μM CPP-KR12 compared to 30 μM KR12 (white arrowheads in Figure 1b). Conversely, *E. coli* treated with 30 μM CPP-KR12 exhibited a significant number of broken and clumped cell shapes.

It is assumed that the enhanced antibacterial activity of CPP-KR12 is attributed to its increased intracellular transport of KR12 via CPP conjugation, given that CPP is able to enter bacteria cells without damaging the membrane and has no antibacterial activity. The membrane permeability of CPP-KR12 to *S. aureus* was lower than that of Gram-negative bacteria, suggesting that the lower permeability of CPP-KR12 is related to the lower antibacterial activity in *S. aureus* (Table 2). KR12-induced pores in *E. coli* indicate that KR12 is delivered into the cell via pore formation across the cell membrane. It has also been shown that membrane disruption by KR12 is the primary mode of action of KR-12 activity against Gram-negative bacteria [33]. Treatment with a concentration of 30 µM CPP-KR12 caused significant changes in cell morphology, as observed in Figure 1b. At low concentrations, CPP-KR12 can enter the cell without causing any damage to the cell membrane. However, at higher concentrations, it may lead to the disruption of the membrane, resulting in cell lysis, as shown in membrane disruption by the carpet mechanism [34].

#### 2.1.3. Comparison of AMP’s DNA Binding Ability

**Figure 2:**
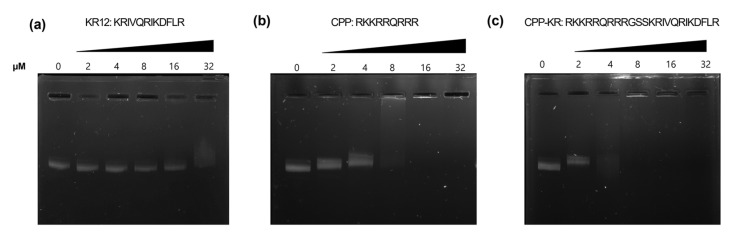
Gel retardation assay of peptide–DNA complexes was performed using peptides of varying concentrations. The peptides used for complexation with DNA were (**a**) KR12, (**b**) CPP, and (**c**) CPP-KR12. The concentration of peptide used for complexation with DNA is expressed in micromolar units.

Certain AMPs have the potential to target intracellular molecules after penetrating the cell membrane. Therefore, we investigated the DNA binding properties of the peptides (Figure 2). Plasmid DNA was combined with various peptide concentrations for 30 min at room temperature (25 °C) to generate complexes referring to the method of Lee et al. [18]. The delay in plasmid DNA migration on an agarose gel was used to assess peptide binding capacity to DNA. KR12 only slightly impacted DNA migration at a concentration of up to 32 µM; however, CPP-KR12 entirely stopped DNA migration at a concentration of 4 µM, indicating that CPP-KR12 has a high affinity for binding to DNA. The strength of this binding is heavily influenced by CPP, which inhibits DNA migration at 8 µM. The net charge of KR12, CPP, and CPP-KR12 is +4, +8, and +12, respectively (as shown in Table 1). The capacity for DNA binding appears to grow proportionally to the rise in cationicity.

CPP-KR12 had a higher membrane permeability in Gram-negative bacteria than KR12 and appeared to be associated with more potent antibacterial effects in Gram-negative bacteria. However, the membrane permeability of CPP itself does not necessarily correlate with antibacterial efficacy, and there are no significant differences in membrane permeability among the peptides in *S. aureus*. This suggests that other targets may be involved in the bactericidal effects of the KR12 and CPP conjugate. Increasing evidence has shown that antimicrobial peptides (AMPs) may also exert intracellular inhibitory activity as a primary or secondary mechanism for more efficient killing [35].

Although CPP, which has a high DNA binding capacity, lacks antibacterial activity and KR12 has a relatively low DNA binding capacity, KR12 may have other intracellular targets for bacterial death. The intracellular targets of LL37 and KR12 are not yet fully understood; however, several studies have identified their antimicrobial mechanisms. *E. coli* was used to express LL37 in order to determine its intracellular targets [36]. Under aerobic growth circumstances, LL37’s effects were investigated, and it was discovered that intracellular LL37 expression boosted the formation of reactive oxygen species (ROS) [36]. This led to lethal membrane depolarization, confirming that ROS production is a bactericidal mechanism for LL37 under such conditions [36]. Another study has also found that LL37 mainly increases the production of oxidative molecules that cause cell damage in both Gram-positive and Gram-negative bacteria [37]. Therefore, the antibacterial activity of CPP-fused KR12 is believed to be enhanced because of increased membrane permeability and affinity for intracellular DNA, in addition to the ROS generation effect of KR12.

#### 2.1.4. Anti-Inflammatory Effect of AMP on Lipopolysaccharide (LPS)-Induced Inflammation

**Figure 3:**
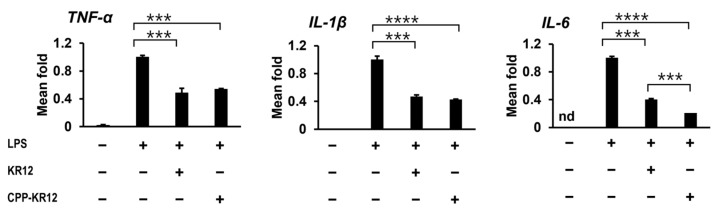
Anti-inflammatory response of AMPs in LPS-stimulated RAW264.7 cells. The mRNA expression levels of inflammatory cytokines, namely *TNF-α*, *IL-1β*, and *IL-6*, were measured by qRT-PCR. The Glyceraldehyde-3-Phosphate-Dehydrogenase (GAPDH) gene was used for normalization in gene expression. The gene levels in each treated cell were calculated relative to those in cells exposed to LPS without AMP. *IL-6* was not detected in the control without both LPS and AMP; therefore, it was indicated as nd (not detected). Values are presented as the mean ± SE (N = 3). *** *p* < 0.001, **** *p* < 0.0001.

To investigate the ability of peptides to neutralize LPS, we measured the mRNA expression levels of LPS-stimulated tumor necrosis factor-alpha (TNF-α), interleukin 1 beta (IL-1β), and interleukin 6 (IL-6) in RAW264.7 macrophages (Figure 3). The expression of these cytokines increased after LPS treatment but decreased by approximately 50% compared to the LPS-induced expression by KR12 and CPP-KR12 treatment. The expression level of IL-6 induced by LPS was lowered more by CPP-KR12 than by KR12.

It has been discovered that some AMPs can successfully neutralize LPS, producing bactericidal and anti-inflammatory actions against Gram-negative bacteria [38,39]. In addition to its antimicrobial properties, AMP’s immunomodulatory abilities in infected tissues are important for healing by restoring the host’s protective immunity [40]. After observing CPP-KR12′s potent antibacterial activity against Gram-negative bacteria, we assumed that it would have a greater impact on reducing LPS-induced inflammation compared to KR12. However, as shown in Figure 3, CPP-KR12 only exhibited increased LPS-neutralizing activity in *IL-6* expression, while no significant difference was observed in *TNF-α* and *IL-1β* expressions when compared to KR12. In addition, this inhibition was not observed in CPP-treated LPS-stimulated RAW 264.7 cells (Supplementary Figure S2), suggesting that KR12 is responsible for the immunomodulation [41].

### 2.2. Self-Entrapment of AMPs in Silica Particles via AMP-Mediated Silica Deposition

Next, KR12 and CPP-KR12 were investigated to determine their ability to synthesize silica and their potential as antibacterial agents. The amount of silica formed per 100 μg of each peptide, the loading efficiency of peptide in silica, and self-entrapment efficiency are shown in Table 3. The order of silica forming ability was CPP-KR12 > CPP > KR-12. Both CPP and CPP-KR12 were entrapped by 95% of the added amount in the as-prepared silica. Co-precipitation of peptide and silica resulted in 70% or higher loading efficiencies. The R5 peptide (SSKKSGSYSGSKGSRRIL) is generated from silaffin peptides naturally found in the diatom *Cylindrotheca fusiformis* and possesses excellent silica precipitation activity [42]. In the presence of negatively charged phosphate ions, the positively charged ε-amino groups of the lysine residues in R5 catalyze the formation of siloxane bonds, resulting in silica deposition from silicic acid (Si(OH)_4_) in vitro [43]. Our previous study demonstrated the efficient delivery of cationic BMP2 protein and P4 peptide to osteoblast via silica nanoparticles formed using BMP2 or P4 peptide-mediated biomimetic silicification methods [44,45]. Here, we have confirmed that the antimicrobial peptides KR12 and CPP-KR12 also possess the capability to undergo silica formation.

**Figure 4:**
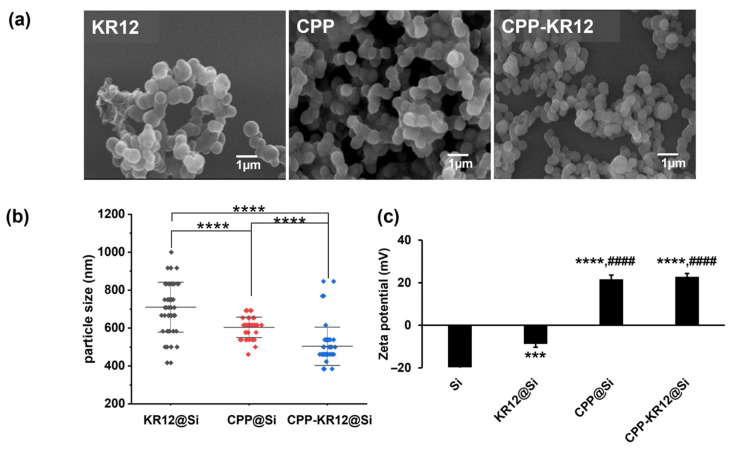
Shape and properties of silica particles formed by each AMP. SEM image at 10,000× magnification (**a**) and size distribution (**b**) of silica particles formed by each AMP. The dispersion of particle size was obtained by measuring the diameter of 50 particles from SEM images. The middle line on the figure represents the average sizes for the corresponding particles and the upper and lower lines represent standard deviation. ****p < 0.0001. (**c**) Zeta potential of each silica particle dispersed in PBS. The zeta potential value is the mean and standard deviation of the three measurements. ***p < 0.001, ****p < 0.0001 vs. Si, #### *p* < 0.0001 vs. KR12@Si.

For CPP and CPP-KR12, silica precipitation occurred as soon as the silica precursor, peptide, and phosphate ion were mixed, and all appeared in particulate form (Figure 4a). In contrast, KR12 formed more slowly than the other peptides, resulting in a mixture of particle and gel-like forms. The average particle sizes of 50 randomly selected particles from SEM images were measured and found to be 700, 600, and 500 nm in the order KR12 > CPP > CPP-KR12 (Figure 4b). The zeta potential of each silica particle was measured by dispersion in phosphate buffered saline (PBS) (Figure 4c). The peptide-free silica particle showed a zeta potential of –19.74 ± 0.91 mV, whereas the silica particles formed by CPP and CPP-KR12 displayed positive values of 21.63 ± 1.91 mV and 22.73 ± 1.58 mV, respectively. In contrast, KR12 exhibited a zeta potential of –8.73 ± 1.60 mV. The zeta potential values indicated the presence of positively charged properties in the particles attributed to the peptide. It is suggested that the CPP or CPP-KR12 exhibited a cationic nature on the surface of the particle rather than being buried within the silica. These zeta potential values indicated the presence of positively charged properties on the particles attributed to the peptides. It is suggested that CPP or CPP-KR12 exhibited a cationic nature on the surface of the particles rather than being buried within the silica. The zeta potential of particles is an important characteristic that influences particle stability, and the cell is an important characteristic that influences particle stability and cell adhesion [46]. The value of zeta potential, whether positive or negative, plays a significant role in particle suspension stabilization [47]. This is because electrostatic repulsion between particles with the same charge produces particle separation. A positive zeta potential value is expected to encourage particles to adhere to negatively charged surfaces more.

There were differences in particle size depending on the peptide, with larger net positive charges tending to have smaller particle sizes (Table 1 and Figure 4a). The anionic orthosilicate strongly binds to the cationic residues of CPP or CPP-KR12, resulting in a high degree of silica particle nucleation, leading to smaller particle sizes. However, in the case of KR12, the cation is dispersed by hydrophobic amino acid residues, resulting in relatively weak binding to the anionic orthosilicate, leading to a lower degree of silica nucleation and larger particle sizes due to processes such as Ostwald ripening [48,49]. Atomistic molecular dynamics simulations revealed that the orthosilicate anion does not bind as strongly to the trimethylated lysine of the R5 peptide, resulting in a lower degree of nucleation of the trimethylated R5, leading to larger particle sizes as compared to that of R5 [48].

### 2.3. Characterization of CPP-KR12 In Silica Nanoparticle Form 

#### 2.3.1. Minimum Inhibitory Concentration of AMP@Si

The antimicrobial activity of AMP-loaded silica particles was compared by determining the MIC (Table 4). The concentration equivalent to free AMP was determined for AMP@Si by calculating the amount of AMP trapped within the silica based on loading efficiency (LE)% and entrapment efficiency (EE)% (Table 3). Compared to the free form, the antibacterial activity of KR12 in silica resulted in almost similar MIC concentrations against both *E. coli* and *P. aeruginosa*. In contrast, it significantly decreased against *S. aureus* (*p* = 0.013, Supplementary Table S2). The antibacterial activity of CPP-KR12-loaded silica particles against both Gram-negative and Gram-positive bacteria showed a slight increase in MIC compared to those of the free forms; however, there were no significant differences (Supplementary Table S2). It may be possible that the silica particulate form of KR12 may provide a greater delivery of KR12 into *S. aureus* compared to the free form, as free KR12 has shown higher MIC against *S. aureus*.

#### 2.3.2. Comparison of CPP-KR12 Delivery between Free and Immobilized Form

**Figure 5:**
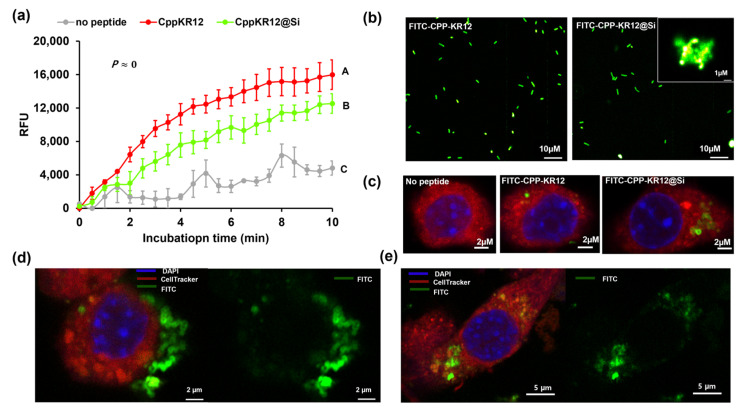
Fluorometric determination of the relative sensitivity of suspensions of *E. coli* to free CPP-KR12 and CPP-KR12@Si with SYTOX^TM^ Green stain. *E. coli* (10^8^ CFU/mL) was incubated with 5μM of SYTOX^TM^ Green at room temperature for 20 min in 1× PBS. The fluorescence emission spectrum of each 490 nm-excited 100-fold diluted *E. coli* suspension in the absence or presence of 30 μM of CPP-KR12 or CPP-KR12@Si was acquired for 10 min (**a**). At the 0.05 level, the mean values for the groups treated with no peptide, CPP-KR12, and CPP-KR12@Si are significantly different from each other (two-way analysis of variance (ANOVA), coupled with Bonferroni post hoc test for multiple comparison). Means that do not share a letter are significantly different. Delivery of FITC-CPP-KR12 to *E. coli* (**b**) and to Raw264.7 cells (**c**). Silica particles were labeled by FITC-conjugated corresponding peptides. Cytosol and nuclei of Raw264.7 were stained by CellTracker Red and 4’,6-diamidino-2-phenylindole (DAPI), respectively. Raw264.7 cells exposed to FITC-CPP-KR12@Si for 2 h (**d**) and 24 h (**e**). In (**d**,**e**), the left panel displays the merged fluorescence of DAPI, CellTracker, and FITC, which helps identify nuclei, cytosol, and particles. In contrast, the right panel only shows FITC fluorescence, emphasizing the distribution of particles.

The SYTOX^TM^ Green uptake assay was used to compare the release of CPP-KR12 from the immobilized form in silica and free forms into *E. coli*. CPP-KR12@Si showed an approximately 30% decrease in cell membrane permeation rate over 10 min compared to CPP-KR12 (Figure 5a). *E. coli* and Raw264.7 cells were observed after treatment with either silica particles formed by FITC-CPP-KR12 or an equivalent amount of FITC-CPP-KR12 based on peptide concentration. The delivery of FITC-CPP-KR12 into *E. coli* was observed in both the free and the immobilized form (Figure 5b). No aggregated *E. coli* fragments were observed in the FITC-CPP-KR12-treated sample; however, aggregated particulate forms with a strong FITC fluorescence signal were observed in the FITC-CPP-KR12@Si-treated sample (Figure 5b). This suggested that the peptide was delivered to the bacteria even in immobilized form.

In the delivery of FITC-CPP-KR12 into Raw264.7 macrophages (Figure 5c), both the free and immobilized forms were delivered to macrophages, and the intracellular FITC signal was detected (Figure 5c). Compared to the free form, the particulate form was delivered more locally to the cells, indicating a more concentrated effect on the cells. The fluorescence analysis conducted by the STEDYCON microscope revealed that FITC-labeled CPP-KR12 was present at the edges of the silica particles, as shown in Figure 5d. Furthermore, the subcellular localization of CPP-KR12@Si was compared over time, and it was observed that after 2 h of exposure the particles were intact and bound to the cell membrane. However, after 24 h they were found inside the cell and in a degraded form, as seen in Figure 5e. This suggests that the silica particles carrying the peptide enter the cell, deliver the peptide, and then degrade.

The distribution of the peptides at the edges of the silica particle was believed to be the cause of the highly cationic nature of CPP-KR12@Si (Figure 4c) and its comparable antimicrobial activity to the free form (Table 2 and Table 4). When *S. aureus* infects, it is phagocytized by macrophages. In this case, the bacteria do not die but survive and persist in the host cells, making it a major cause of post-operative infections. These cells can act as reservoirs, leading to chronic infections [13]. TAT-KR12 has been reported to enter cells and inhibit the growth of intracellularly infected *S. aureus* [13]. Similarly, CPP-KR12@Si might be able to penetrate infected cells and interfere with their intracellular survival.

#### 2.3.3. Stability of AMP@Si against Protease Treatment

**Figure 6:**
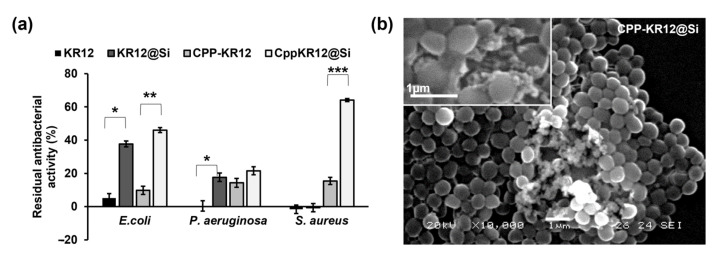
Comparison of residual antibacterial activity of AMP and AMP entrapped in silica after trypsin treatment and SEM image of *S. aureus* treated with CPP-KR12@Si. (**a**) The residual antibacterial activity was calculated by taking the difference in optical density at 600 nm between the negative control and the sample containing the trypsinized AMP. It was then divided by the optical density of the negative control and multiplied by 100. Each bacterial solution grown in a medium without AMP is used as a corresponding negative control. Values are presented as the mean ± SE (N = 3). * *p* < 0.05, ** *p* < 0.01, or *** *p* < 0.001 vs. corresponding free form of AMP in each strain. (**b**) SEM image of *S. aureus* treated with CPP-KR12@Si. The image is a 10,000× magnification, the inside image is a 20,000× magnification, and the scale bar represents 1 µm.

The absorbance value of the bacteria in the absence of AMP was used as the negative control. The absorbance value of the bacteria grown in the presence of trypsinized AMP was subtracted from that of the negative control, and the resulting difference was expressed as a percentage fraction of the negative control, yielding the residual antibacterial activity of AMP after trypsin treatment (Figure 6a). Free KR12 lost 100% of its antibacterial activity against *P. aeruginosa* and *S. aureus* after trypsin treatment. It showed approximately 5% residual activity against *E. coli*. CPP-KR12 showed approximately 10% residual antibacterial activity against *E. coli* and approximately 15% residual activity against *P. aeruginosa* and *S. aureus*. Silica particulate AMPs also retained antibacterial activity after trypsin treatment, with KR12@Si showing 40% and CPP-KR12@Si showing 45% residual antibacterial activity against *E. coli*. In *P. aeruginosa*, KR12@Si showed 18% and CPP-KR12@Si showed 20% residual antibacterial activity. In *S. aureus*, KR12@Si lost its antibacterial activity, whereas CPP-KR12@Si retained more than 60% of its antibacterial activity. When *S. aureus* was treated with CPP-KR12@Si, aggregation was not seen with free AMP. SEM images of CPP-KR12@Si-treated *staphylococci* showed the presence of silica particles between their aggregates and the absence of the bacteria (Figure 6b) or membrane disruption around the silica particles (Figure 6b inside). It has been suggested that the silica particles promote aggregation of *S. aureus* and release antimicrobial peptides at the aggregation site, causing the bacteria to rupture their membranes and die (Figure 6b). The localized aggregation of the silica particles and the release of the antimicrobial peptide were assumed to be responsible for the increased killing of *S. aureus* compared to the free form.

Despite their excellent antimicrobial activity under laboratory conditions and consideration of alternatives to conventional antibiotics, AMPs have been limited in clinical use because of their loss of activity via rapid degradation by plasma and bacterial proteases and their short half-life in the body resulting from rapid hepatic and renal elimination [50]. Several techniques have been employed to improve the stability of AMPs. These strategies involve substituting L-amino acid residues with D- and unnatural amino acids, modifying the N- and/or C-terminus, cyclizing the peptide, using non-peptidic backbones (peptidomimetics), and multimerizing AMP monomers [50,51,52,53,54]. Meanwhile, silica particles offer advantages as a delivery system for AMPs, such as a controlled release rate, prevention of protein degradation, and inhibition of binding to serum proteins [55,56], which can prolong the circulation of AMPs in the bloodstream and increase their bioavailability [17].

### 2.4. Cytotoxicity and Hemolytic Activity of AMPs and AMP@Sis

**Figure 7:**
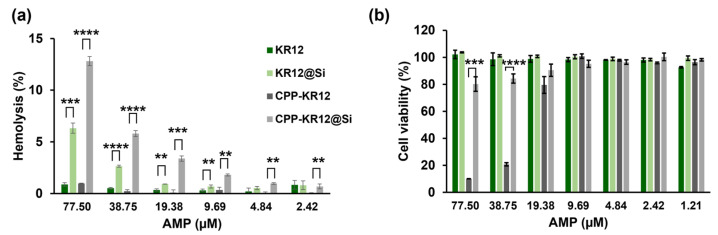
The hemolytic activity and cytotoxicity of AMPs. (**a**) Hemolysis (%) is the difference in optical density at 570 nm of sheep red blood cells (RBCs) between samples containing the indicated AMP and the negative control as a percentage of the difference in optical density between the positive and negative controls. RBCs present in PBS alone serve as a negative control, whereas RBCs treated with 1% Triton X-100 in PBS serve as a positive control. Values are presented as the mean ± SE (N = 3). ** *p* < 0.01, *** *p* < 0.001, or **** *p* < 0.0001 vs. corresponding free form of AMP in each concentration. (**b**) The cytotoxicity of AMP was measured 24 h after the addition of each indicated concentration of AMP to Raw264.7 cells grown overnight. Cell survival is expressed as a percentage of the negative control grown without AMP. Values are presented as the mean ± SE (N = 3). *** *p* < 0.001 or **** *p* < 0.0001 vs. corresponding free form of AMP in each concentration.

The hemolytic activity (Figure 7a) and cytotoxicity against mammalian cells (Figure 7b) of free AMP and AMP immobilized on silica were compared. KR12 and CPPKR12 showed no significant hemolytic activity against sheep red blood cells (RBCs), with less than 1% hemolysis at concentrations up to 77.5 μM. However, at the same concentration, KR12@Si and CPP-KR12@Si significantly increased the hemolytic activity to 6.3% and 12.8%, respectively. Hemolytic activity below 1% was observed at 19.38 μM for KR12@Si and 4.84 μM for CPP-KR12@Si. Higher hemolytic activity was observed for silica particles synthesized without the peptides, suggesting that the erythrocyte cell membrane is more sensitive to silica nanoparticles than to AMP itself, resulting in increased hemolysis in the silica particle form compared to the free form.

The study found that KR12 showed no cytotoxicity on day 1 of AMP-treated Raw264.7 cells at concentrations up to 77.5μM, as observed in erythrocyte hemolysis assay. Similarly, KR12@Si showed no cytotoxicity up to 77.5μM. In contrast, CPP-KR12 exhibited only 10% cell viability at a concentration of 77.5 μM and 80% cell viability at a concentration of 19.38 μM. Interestingly, CPP-KR12@Si exhibited 80% cell viability at a concentration of 77.5 µM, indicating that immobilization of AMP on silica can reduce the toxicity of CPP-KR12. We have conducted an analysis to demonstrate that silica-entrapped CPP-KR12 has a lower level of cytotoxicity compared to free CPP-KR12. By extrapolating the half maximal inhibitory concentration (IC50) values from Figure 7b, we found that the IC50 of CPP-KR12 was 25.6 ± 7.7 µM and the IC50 of CPP-KR12@Si was 133 ± 24.9 µM. Our results indicate that CPP-KR12 entrapped in silica led to an approximately 5-fold decrease in IC50 compared to the free form. Therefore, we suggest that CPP-KR12@Si has reduced cytotoxicity in comparison to the free form.

Although hemolysis refers primarily to the destruction of RBCs, cytotoxicity refers to the decreased viability of cells. Therefore, even if a substance destroys RBCs and causes hemolysis, there may be no cytotoxicity if the substance acts only on the outside of the cell and does not penetrate the cell. It is also possible that a substance does not cause membrane disruption on the outside of the cell; however, once inside the cell it causes cytotoxicity by acting on intracellular targets. Based on the results, CPP-KR12 is more likely to cause cytotoxicity inside the cell than at the cell membrane. According to a study by Yu et al. [57], SiO_2_ cytotoxicity depends on the cell type and is also affected by surface charge and pore size. After 24 h of incubation, they found that non-porous or mesoporous SiO_2_ induced acute toxicity in RAW 264.7 macrophages, resulting in only 20–40% of viable cells compared to the control, whereas amine-modified SiO_2_ exhibited minimal toxicity, leaving 64–85% of viable cells. They postulated that the silanol groups on the surface of anionic silica particles were toxic because of their strong affinity for the plasma membrane. The shielding of the surface silanol groups by amine functionality reduced the cellular accessibility of the silanol groups, thus decreasing cytotoxicity [58,59]. It is also believed that CPP-KR12@Si shares characteristics with amine-functionalized silica particles.

CPP-KR12 exhibited a higher degree of internalization compared to KR12, which could potentially lead to intracellular toxicity. The transcription factor nuclear factor erythroid 2-related factor 2 (NRF2) can regulate the expression of antioxidant proteins such as superoxide dismutase (SOD) and glutathione peroxidase (GPx), which can scavenge ROS and protect cells from damage [60]. In Raw264.7 cells without LPS, CPP-KR12 treatment increased the mRNA expression levels of NRF-2, SOD1, and GPx1, indicating increased ROS generation. KR12 is known to exert antimicrobial properties via ROS generation (Supplementary Figure S3) [36,37]. The treatment of LPS or KR12 resulted in a decrease of approximately 50% in the expression levels of antioxidant proteins in Raw264.7 cells compared to the control. However, the concentration of CPP-KR12 used in the experiment (6 µM) was not cytotoxic. This suggests that the increase in mRNA levels of antioxidant proteins may be a protective mechanism activated by CPP-KR12 (Supplementary Figure S3). It is worth noting that NRF-2 is considered as a double-edged sword [61]. Therefore, CPP-KR12 can induce cytotoxicity in mammalian cells at high concentrations; however, at low concentrations it may have immunomodulatory and cytoprotective effects. At high concentrations, CPP-KR12’s increased permeability and DNA binding capacity, combined with intracellular ROS production, may lead to potential cytotoxicity. In contrast, the cytotoxicity of CPP-KR12 was found to be reduced when it was entrapped in silica, as its intracellular delivery was delayed. After a 5-day incubation period, both the free and immobilized forms showed similar cell viability at 77.5 μM, with a 40% survival rate (Supplementary Figure S4). The increased cell viability observed in the free form on day 5 seemed to be because of the growth of surviving cells. In summary, when delivered in a silica-entrapped form, it becomes more resistant to proteases and is less cytotoxic while retaining its antimicrobial activity.

### 2.5. AMP-Device Combination Products

**Figure 8:**
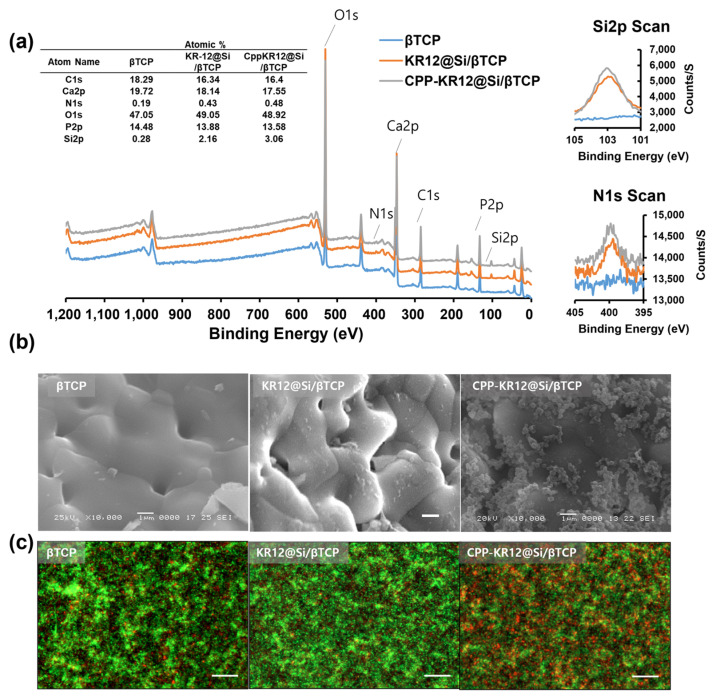
AMP-device combination and its antimicrobial activity. (**a**) X-ray photoelectron spectroscopy of the as-prepared carriers. Atomic percentages of main elements observed in carriers. The atomic percentages of the constituent were calculated as the ratio of each atom to the sum of the main compounds, except carbon. (**b**) SEM images of AMP-device combination. The image is a 10,000× magnification. Scale bar: 1 μm. (**c**) Fluorescence microscope images of *E. coli* stained with BacLight live/dead kit, which displays green for the live bacteria and red for dead bacteria in medium solutions with the indicated device. Scale bar: 20 μm.

To determine the surface composition of the AMP@Si-coated BGS that is based on beta-tricalcium phosphate (β-TCP), X-ray photoelectron spectroscopy (XPS) was employed. Survey spectra are typically recorded in the broad binding energy range of 0 to > 1000 eV for elemental analysis to collect the signatures of all species present on the surface of samples [62]. XPS survey scan spectra exhibited the silica peak at 154 and 103 eV and the nitrogen peak at 398.08 eV in both KR12@Si/BGS and CPP-KR12@Si/BGS (Figure 8a). When comparing the atomic ratios among the BGSs (Figure 8a), it was evident that the surface coating decreased the atomic ratios of Ca and P while increasing the atomic ratios of O, N, and Si. This indicates the presence of silica (SiO_2_) and peptide in both the KR12@Si- and CPP-KR12@Si-coated BGSs. SEM images revealed that the control had a smooth surface, whereas the KR12@Si-coated surface had a thin film of silica and the CPP-KR12@Si-coated surface had a particulate silica coating (Figure 8b). XPS analysis showed little difference in composition, but differences in surface morphology were observed because of differences in the silica deposition patterns of KR12 and CPP-KR12.

To evaluate the efficacy of the antimicrobial BGS combination, *E. coli* was inoculated with the formulated BGS in the medium and incubated overnight. The antibacterial activity of AMP@Si/BGS was determined using Live/Dead^TM^ BacLight™ bacteria viability kits (ThermoFisher Scientific Inc., Waltham, MA, USA). Regardless of the integrity of the cell membrane, SYTO 9 enters all cells and binds to DNA and RNA, generating green fluorescence [63]. In contrast, propidium iodide (PI) only enters cells with damaged cell membranes and binds to nucleic acids, generating red fluorescence [64]. As PI has a higher affinity for nucleic acids than SYTO9, PI will bind to nucleic acids instead of SYTO9 when DNA is exposed to the two dyes at the same time [65]. Based on the combination of these two DNA binding and membrane permeability dependent stains, the red signal is considered as non-viable cells and the green signal represents viable cells [63]. Although *E. coli* exposed to control or KR12@Si/BGS showed similar viability with a high ratio of live bacteria, many dead cells stained red were observed in CPP-KR12@Si/BGS, indicating that CPP-KR12@Si/BGS exhibited effective antibacterial activity compared to KR12@Si/BGS. It has been reported that the release rate of biomolecules immobilized on gel-like silica was slower than that on particulate silica [66]. Therefore, the difference in the silica formulation and the lower cell permeability compared to CPP-KR12 was assumed to be why KR12@Si/BGS did not exhibit antibacterial activity in the current formulation. The loading efficiency of CPP-KR12 per BGS was determined to be 0.1%. This indicates that 50 µg of AMP was loaded into 50 mg of BGS per 1 mL of medium. Converting this to a molar concentration yields an AMP concentration of approximately 16 µM. This concentration is close to the MIC for *E. coli* (14.54 µM), but it is not toxic to eukaryotic cells. The antimicrobial bone graft device was prepared by coating CPP-KR12 in the form of silica particles on the surface of bone graft material. This showed an antimicrobial effect at non-toxic doses to host cells. In the future, we will evaluate whether this composite not only delivers antimicrobial agents locally but also exerts a positive effect on promoting bone regeneration by the parallel immobilization of bone regeneration-promoting factors on silica.

## 3. Materials and Methods

### 3.1. Materials

The peptides named KR12 and CPP-KR12 were synthesized by GenScript (Piscataway, NJ, USA) and CPP peptide and fluorescein isothiocyanate (FITC)-labeled CPP-KR12 was synthesized by Peptron (Peptron, Dajeon, Republic of Korea). FITC was conjugated to the N-terminus of the peptide through a 6-aminohexanoic acid (Ahx) linker. After purification by HPLC and subsequent LC/mass analysis, the synthetic peptides used in this work were received from each provider. The peptides had a purity of 95%. Table 1 shows the obtained mass data as well as the peptide sequences. Tetramethyl orthosilicate (TMOS), L-ascorbic acid, ammonium heptamolybdate, and Lipopolysaccharides (LPS) from *Escherichia coli* O111:B4 were purchased from Sigma-Aldrich (St. Louis, MO, USA). Uncoated granule type BGS made of β-TCP was provided by CG-Bio (CG Bio Inc., Seoul, Republic of Korea), which is granule-type BGS with sizes ranging from 0.6 to 1 mm and average 300 μm-sized interconnected pores and porosity of 70–80% [24]. The Pierce Quantitative Fluorometric Peptide Assay Kit, CellTracker Red CMTPX (Invitrogen, Waltham, MA, USA), SYTOX^TM^ Green Nucleic Acid Stain, and Live/Dead^TM^ BacLight^TM^ Bacterial Viability Kits were purchased from Thermo Fisher Scientific Inc. (Thermo Fisher Scientific Korea, Seoul, Republic of Korea). The CellTiter 96^®^ AQueous One Solution Cell Proliferation Assay (MTS) was purchased from Promega (Promega Korea Ltd., Seoul, Republic of Korea). All other reagents were of analytical grade.

### 3.2. Bacterial Strains

Two Gram-negative bacterial strains, namely *Escherichia coli* (wild type W2110) and *Pseudomonas aeruginosa* (wild type PK01) and Gram-positive strain, *Staphylococcus aeruginosa* (ATCC 29213) were obtained from the Department of Biotechnology and Bioinformatics at Korea University.

### 3.3. Minimum Inhibitory Concentration (MIC) of Antimicrobial Peptides (AMPs)

The broth microdilution assay was used to test the antimicrobial activity of AMPs, as previously reported [67]. Briefly, bacteria were grown to mid-logarithmic growth phase in Mueller Hinton Broth (MHB) media at 37 °C. Then, in 96-well plates, 50 µL of peptide solution (0–320 µM) diluted using a 2-fold serial dilution method was mixed with an equal volume of bacterial solution (3 × 10^6^ CFU/mL) and incubated for 18 h at 37 °C with shaking at 200 rpm. The negative control was the culture medium itself. The lowest antimicrobial peptide concentration that exhibited the same turbidity (<0.1) as the negative control after measurement with a microplate reader (Infinite M200 PRO NanoQuant; TECAN, Männedorf, Switzerland) at 600 nm was considered the MIC. The assay was performed in triplicate.

### 3.4. Silica Deposition and Quantification

According to Luckarift et al. [19], AMP-catalyzed silica deposition was started by adding 10 µL of 0.5 M sodium phosphate (pH 7.6) buffer to 90 µL ddH_2_O containing 100 µg of AMP and 10 µmoles of hydrolyzed TMOS. The reaction was performed at room temperature (25 °C) for 5 min with stirring. The silica precipitate was washed by centrifugation three times with ddH_2_O (14,000× *g*, 5 min, 4 °C). Silica precipitation was determined using a modified molybdenum blue assay, as previously described [45]. The washed silica precipitate was dried, dissolved in 1 M NaOH solution, and diluted 5- to 10-fold. The total reaction solution was 50 μL. Equal volumes of 1% ammonium heptamolybdate, 1% oxalic acid, and 100 mM ascorbic acid were added sequentially to this mixture. Optical density was measured at 810 nm.

### 3.5. Measurement of Entrapping and Loading Efficiency of AMP in Silica Particles

As described above, a total of 100 μg of peptide was used for the precipitation of silica particles loaded with the corresponding peptides. The amount of co-precipitated peptide was estimated by subtracting the amount of AMP that remained in the supernatant after the precipitates were removed from the original feeding amount. The quantification of peptide was determined using the Pierce Quantitative Fluorometric Peptide Assay kit (Thermo Fisher Scientific, Waltham, MA, USA). The amount of silica was determined using the above-mentioned modified molybdenum blue method. The entrapment efficiency (%) and loading efficiency (%) of AMP were estimated using the following equations:

(1) $$Entrapment\;Efficiency\left(EE\right)\;\left(\%\right)=\frac{mass\;of\;bound\;AMP}{initial\;mass\;of\;AMP\;added}\times 100$$

(2) $$Loading\;Efficiency\left(LE\right)\%=\frac{mass\;of\;bound\;AMP}{total\;mass\;of\;delivery\;system}\times 100$$

### 3.6. SYTOX^TM^ Green Uptake Assay

SYTOX^TM^ Green Nucleic Acid Stain was used to fluorometrically determine the relative sensitivity of *E. coli*, *P. aeruginosa*, and *S. aureus* suspensions to different AMPs [68]. The relative sensitivity of free CPP-KR12 and CPP-KR12@Si to *E. coli* suspension was also determined fluorometrically by SYTOX Green staining. *E. coli* (10^8^ CFU/mL) was incubated with 5 μM SYTOX Green in 1× PBS at room temperature for 20 min, and a 100-fold diluted *E. coli* suspension was recorded in the absence or presence of 30 µM CPP-KR12 or CPP-KR12@Si for 10 min. The fluorescence emission spectrum was excited at 490 nm.

### 3.7. Stability of AMP@Si against Protease Attack

Free AMP or AMP@Si was dissolved in 1 × PBS at a concentration of 300 μM and left at 37 °C for 3 min in the presence of 0.025% trypsin, followed by the addition of fetal bovine serum (FBS) to stop the activity of trypsin. Bacteria were incubated overnight with each trypsinized peptide formula, and broth absorbance was measured at 600 nm. As a negative control, the absorbance of the strain grown in the absence of the peptide was set. To determine the remaining antimicrobial activity of AMP after treatment of trypsin, the absorbance of the sample treated with AMP was subtracted from the absorbance of the negative control. The resulting difference was then expressed as a percentage of the absorbance of the negative control.

### 3.8. Cytotoxicity and Hemolytic Activity Assay

Free peptides and silica particles loaded with them (AMP@Si) were compared for their cytotoxicity against mouse macrophages (Raw264.7 cells) and their hemolytic activity against RBCs. Free AMP or AMP@Si was added to each well of a 96-well plate in 2-fold serial dilutions to 50 μL with DMEM medium containing 10% FBS and 1% antibiotics. Raw264.7 macrophages at a concentration of 4 × 10^4^ cells per 50 μL were added to the wells containing the peptide to give a final volume of 100 μL. Cell proliferation was confirmed by MTS assay (Promega, Madison, WI, USA) after 24 h or 5 days of incubation. The MTS tetrazolium compound was reduced by viable cells to generate a colored formazan product. The intensity of the color at 490 nm is proportional to the number of viable cells in the sample. To calculate cell viability, the absorbance of cells treated with the peptide was divided by the absorbance of cells that were not treated with the peptide. The resulting quotient was then expressed as a percentage. Fresh RBCs from sheep blood (KisanBio, Seoul, Republic of Korea) were recovered by centrifugation at 150× *g* for 5 min and washed three times with PBS. Peptides and silica particles diluted in 2-fold serial dilutions with PBS were added to each well of a 96-well plate, giving a total volume of 100 μL. A 2% RBCs suspension (2% *w*/*v* RBC in PBS) was added to each concentration of peptide in 100 μL to give a final volume of 200 μL, and the RBCs was incubated at 37 °C for 1 h. The supernatant (100 μL) was collected after centrifugation at 1000× *g* for 5 min, and the absorbance (A) was measured at 570 nm [67]. The value of “zero hemolysis” was determined by the absorbance of PBS alone, and 100% hemolysis was determined by the absorbance of the supernatant obtained from 1% (*v*/*v*) Triton X-100-treated RBCs. The percentage of hemolysis was calculated as hemolysis (%) = (A_AMP_ − A_PBS_)/(A_Triton_ − A_PBS_) × 100.

### 3.9. Effect of AMPs on mRNA Expression of Pro-Inflammatory Cytokines 

Quantitative PCR (qPCR) was performed as previously described [44] to evaluate the mRNA expression of pro-inflammatory cytokines, including TNF-α (forward: ATGGCCTCCCTCTCATCAGT; reverse: TGGTTTGCTACGACGTGGG), IL-1β (forward: TGCCACCTTTTGACAGTGATG; reverse: AAGGTCCACGGGAAAGACAC), and IL-6 (forward: GTCCTTCAGAGAGATACAGAAACT; reverse: AGCTTATCTGTTAGGAGAGCATTG) in Raw264.7 cells in the absence or presence of 100 ng/mL LPS. The amount of target genes was expressed as relative quantification (RQ) over the expression level of LPS alone-treated cells. The quantitative gene expression data were normalized to the expression levels of Glyceraldehyde-3-phosphate dehydrogenase (GAPDH) (forward: CCTGGCCAAGGTCATCCATG; reverse: GCAGGAGACAACCTGGTCCT) [69].

### 3.10. Gel Retardation Assay

Gel retardation assays were used to determine the ability of peptides to bind and interact with DNA, following the method of Lee et al. [14]. In brief, increasing amounts of peptides were mixed with 200 ng plasmid DNA (pQE80L) in reaction buffer, which contained 10 mM Tris-HCl, 5% glycerol, 50 µg/mL bovine serum albumin, 1 mM dithiothreitol, and 1 mM ethylenediaminetetraacetic acid (EDTA). As negative controls, wells containing only plasmid were used. The peptide–plasmid mixture was incubated for 30 min in a 37 °C water bath before being separated by agarose gel electrophoresis (1% agarose) in 0.5% TAE buffer. Migration of DNA was detected by RedSafe™ Nucleic Acid Staining Solution (iNtRON Biotechnology, Inc., Seongnam, Republic of Korea).

### 3.11. STED Microscopy and Confocal Images of Cells

Raw264.7 cells on a coverslip were incubated in DMEM with free FITC-CPP-KR12 or FITC-CPP-KR12@Si in a humidified atmosphere containing 5% CO_2_ at 37 °C for 2 h or overnight. The cells were rinsed three times with PBS and fixed with 4% paraformaldehyde at 37 °C for 30 min. After three washes with PBS, cells were immersed in PBS with CellTracker Red CMTPX (Thermo Fisher Scientific) (5000:1 dilution) for 30 min for cytoplasm staining. The stained cells were washed three times with PBS and stained with 1 µg/mL of DAPI (4’,6-diamidino-2-phenylindole) to detect nuclei. The logarithmic growth of *E. coli* was washed three times with PBS and exposed to each peptide formula for 20 min to 1 h. After three PBS washes, the cells were then fixed with 2.5% glutaraldehyde and observed under a fluorescence microscope. Confocal images were taken along the XY plane using a STEDYCON microscope (Abberior Instruments GmbH, Göttingen, Germany) with a 100× oil immersion objective. Raw264.7 cells show rhodamine-labeled cytoplasm (red), FITC-labeled CPP-KR12 or CPP-KR12@Si (green), and DAPI-labeled nucleic acids (blue). *E. coli* shows green fluorescence because of FITC-labeled AMP. The laser power was set to 10% and the pixel size was 30 nm.

### 3.12. Live/Dead Cell Assay

A fluorescence microscope (CytationTM 7, BioTek, Winooski, VT, USA) was used to observe the results of the BacLight live/dead bacterial viability kit according to the manufacturer’s method (ThermoFisher Scientific Korea, Seoul, Republic of Korea). *E. coli* grown overnight was added to the culture medium containing the prepared AMP@Si/β-TCP BGS (50 mg/mL) at a concentration of 100-fold dilution. An equal amount of *E. coli* inoculated into media containing β-TCP without AMP was used as a control to compare antibacterial activity. After incubation at 37 °C for 12 h, the grown *E. coli* was recovered by centrifugation and washed with PBS to remove medium components, then SYTO 9 (3.34 mM) and PI solutions (20 mM) were mixed in an equal 1:1 volume ratio and 3 µL of the mixture was added per 1 mL of the washed bacterial suspension and stained under dark conditions for 15 min. An amount of 5 µL of the stained *E. coli* suspension was carefully applied to a microscope slide. A coverslip was then placed over the sample. The sample was observed under a fluorescence microscope.

### 3.13. Zeta Potential Measurement

For the zeta potential studies, AMP@Si samples were suspended in PBS. The suspensions were injected into zeta cells and allowed to equilibrate at 25 °C for 15 min, and measurements were acquired on a Zetasizer Nano ZS ZEN3600 (Marvern Instrument Ltd., Worcestershire, UK). Zeta potential was calculated from the mean of 10 measurements using the Smoluchowski equation as described previously [70]. Experiments were performed in triplicate.

### 3.14. Sample Preparation of Bacterial Cells for SEM

Mid-logarithmic *E. coli* or *S. aureus* cells (1 × 10^8^ CFU/mL) were cultured in 3 mL of culture medium at 37 °C for 6 h in the presence or absence of AMP. The bacterial suspension was centrifuged, washed three times with PBS, and fixed overnight at 4 °C with 1.5 mL of PBS containing 2.5% glutaraldehyde [67]. After fixation, the bacterial cells were washed three times with sterile ultrapure water. The resulting cells were subjected to a graded ethanol series (30, 50, 70, 80, 90, 95, and 100%) at 25 °C for 10 min each. A smear of 10 µL of the bacterial suspension in 100% ethanol was applied to a glass slide and allowed to air dry.

### 3.15. Preparation of BGS Coated with AMP@Si (AMP@Si /β-TCP)

A 500 mg sample of granular β-TCP, having a size of 0.6 to 1 mm, was added to 1 mL of PBS containing 1 mg of AMP and 20 μmol of hydrolyzed TMOS and gently agitated with a rotary mixer overnight at 4 °C, followed by 10 washes with ddH_2_O to remove unreacted TMOS and peptides. The final BGS was dried in a biosafety cabinet. All procedures were performed under aseptic conditions.

### 3.16. Scanning Electron Microscopy (SEM)

The dried sample was coated with platinum using an ion coater (COXEM Co., Ltd. Daejeon, Republic of Korea) for SEM observation. The structure of bacteria, AMP@Si, and BGS were analyzed using SEM (JSM-6700F, JEOL, Tokyo, Japan).

### 3.17. High-Performance X-ray Photoelectron Spectrometer

The surface chemical compositions of as prepared β-TCP BGS were analyzed by a high-performance X-ray photoelectron spectrometer (HP-XPS, Thermo Scientific ESCALAB 250) with an Al-Kα X-ray source at the Busan Center of the Korea Basic Science Institute.

### 3.18. Statistical Analysis

Statistical analyses were performed using Student’s *t*-test to determine the differences between the two groups. For multiple comparison, two-way ANOVA, coupled with Bonferroni post hoc test, was performed. Statistical significance was set at *p* < 0.05. Statistical analyses were conducted using IBM SPSS Statistics 26 (IBM, Armonk, NY, USA) or Microsoft Excel^®^ LTSC MSO (Microsoft, Redmond, WA, USA).

## 4. Conclusions

The CPP-KR12 peptide is a potent antimicrobial with broad-spectrum activity. Compared to KR12, CPP-KR12 has enhanced cell permeability and DNA binding, resulting in greater antimicrobial activity. Additionally, it can reduce inflammation via LPS neutralization. When CPP-KR12 was mineralized with silica with a 95% entrapment efficiency because of its excellent silica precipitation ability, it showed more resistance to protease attack and reduced cytotoxicity toward Raw264.7 macrophage while retaining antimicrobial activity compared to the free form of CPP-KR12. This silica-mineralized CPP-KR12 (CPP-KR12@Si) binds to the surface of *S. aureus* and induces aggregation, leading to cell death more effectively than the free form, which exhibited low antibacterial activity against the corresponding strain. Despite its potent antimicrobial properties, CPP-KR12 is not cytotoxic at minimally inhibitory concentrations for bacteria. Finally, bone grafts treated with CPP-KR12 as silica particles were shown to be effective against *E. coli* infections. Overall, CPP-KR12@Si is a highly versatile and effective tool in the fight against microbial infections.

## Figures and Tables

**Table 1:** Peptide sequences and their characteristics.

Name	Sequence (N-C)	AA #	Calculated Mass (Da) ^a^	Observed Mass (Da) ^b^	pI ^a^	Net Charge ^a^
KR12	KRIVQRIKDFLR	12	1612.00	1612.80	12.79	+4
CPP	RKKRRQRRR	9	1379.89	1380.00	14.00	+8
CPP-KR12	RKKRRQRRRGSSKRIVQRIKDFLR	24	3163.94	3165.00	13.39	+12
FITC-CPP-KR12	FITC-Ahx-RKKRRQRRRGSSKRIVQRIKDFLR	24	3624.05	3625.92	13.39	+12

^a^ The calculated mass, pI value and net charge were obtained from Pepdraw (https://pepdraw.com/, accessed on 11 September 2023). ^b^ The observed mass was obtained by mass spectrum analysis by company (KR12 and CPP-KR12 from Genscript USA Inc. (Piscataway, NJ, USA) and CPP and FITC-CPP-KR12 from Peptron Inc. (Daejeon, Republic of Korea)). The N-terminus of each peptide was acetylated and the C-terminus was amidated. KR12 and CPP consist of residues 18 to 29 of the human cathelicidin LL-37 [25,26] and residues 49 to 57 (protein transduction domain) of the HIV TAT proteins [27,30], respectively. #: number of amino acid sequences; Ahx: 6-aminohexanoic acid; FITC: Fluorescein isothiocyanate.

**Table 2:** Minimum inhibitory concentration (MIC) (µM) of peptides.

Peptide	*E. coli*	*P. aeruginosa*	*S. aureus*
KR12	103.36 ± 25.82	181.98 ± 68.20	>320 ^a^
CPP-KR12	12.09 ± 4.97	6.12 ± 3.72	22.80 ± 8.48
^b^*p* value	0.248	0.035	0

Minimum inhibitory concentration (MIC) was calculated from three replicate experiments, and values are expressed as the mean ± standard error. The unit is micromoles/L (µM). CPP did not exhibit antibacterial activity against three kinds of bacteria at the highest concentration of 320 µM. ^a^ KR12 did not have antibacterial activity against *S. aureus* at a concentration of 320 µM. ^b^ The *p*-value was calculated by Student’s *t*-test between the MIC of KR-12 vs. the MIC of CPP-KR12 against each strain.

**Table 3:** Comparison of LE% and EE%.

	Initial AMP (μg)	Entrapped AMP (μg)	Silica Deposition (μg)	LE ^a^ (%)	EE ^b^ (%)
KR12@Si	100	56.70 ± 4.71	25.14 ± 2.08	69.16	56.70
CPP@Si	100	95.45 ± 2.06	30.29 ± 2.44	76.31	95.45
CPP-KR12@Si	100	94.45 ± 1.93	33.70 ± 2.86	73.83	94.45

^a^ LE is the peptide loading efficiency, which is defined as the percentage of the amount of peptide in the particle to the total amount of peptide combined with the silica particle. ^b^ EE is the peptide entrapment efficiency, defined as the percentage of the amount of peptide in the particle to the total amount of peptide initially applied to the formulation.

**Table 4:** MIC (µM) of AMP@Si.

Peptide	*E. coli*	*P. aeruginosa*	*S. aureus*
KR12@Si	105.27 ± 2 8.62	>180 ^a^	58.16 ± 19.39
CPP-KR12@Si	14.54 ± 4.89	8.4 0 ± 2.81	28.75 ± 16.87
^b^*p* value	0	0	0.1599

MIC was calculated from three replicate experiments, and values are expressed as the mean ± standard error. The unit is micromolesL^−1^ (µM). ^a^ KR12@Si did not exhibit antibacterial activity against *P. aeruginosa* at the highest concentration of 180 µM. ^b^ The *p*-value was calculated by Student’s t-test between the MIC of KR-12@Si vs. the MIC of CPP-KR12@Si against each strain.

## Data Availability

Data are contained within the article and Supplementary Materials.

## Supplementary Materials

The following supporting information can be downloaded at: https://www.mdpi.com/article/10.3390/ijms242216423/s1.
