# Supplementary material for: Self-Entrapment of Antimicrobial Peptides in Silica Particles for Stable and Effective Antimicrobial Peptide Delivery System
Source: Int J Mol Sci. 2023 Nov 16;24(22):16423. doi: 10.3390/ijms242216423 (PMC10671715; doi:10.3390/ijms242216423)
Supplement: Supplementary file 1 [file ijms-24-16423-s001.zip › ijms-2677635-supplementary.pdf]

Supplementary Materials

Self-entrapment of antimicrobial peptides in silica particles for stable and effective antimicrobial peptide delivery system

Mi-Ran Ki [1,2,*], Tae In Park[1], Sung Ho Kim[1], and Seung Pil Pack [1,*]

[1]Department of Biotechnology and Bioinformatics, Korea University, Sejong-Ro 2511, Sejong 30019, Republic of Korea;

[2]Institute of Industrial Technology, Korea University, Sejong-Ro 2511, Sejong 30019, Republic of Korea;

*Correspondence: MR Ki, allheart@korea.ac.kr, and SP Pack, spack@korea.ac.kr, Tel.: +82-44-860-1419

Table S1. Antimicrobial peptides (AMPs) and their delivery systems used this study

Type	Nomenclature	Description
**AMPs**	KR12	Free form of AMPs
	CPP-KR12	
**AMPs@Si**	KR12@Si	AMPs embedded in silica particles
	CPP-KR12@Si	
**AMPs@Si/BGS**	KR12@Si/β-TCP	AMPs-device combination with β-TCP used as bone graft substitute
	CPP-KR12@Si/β-TCP	

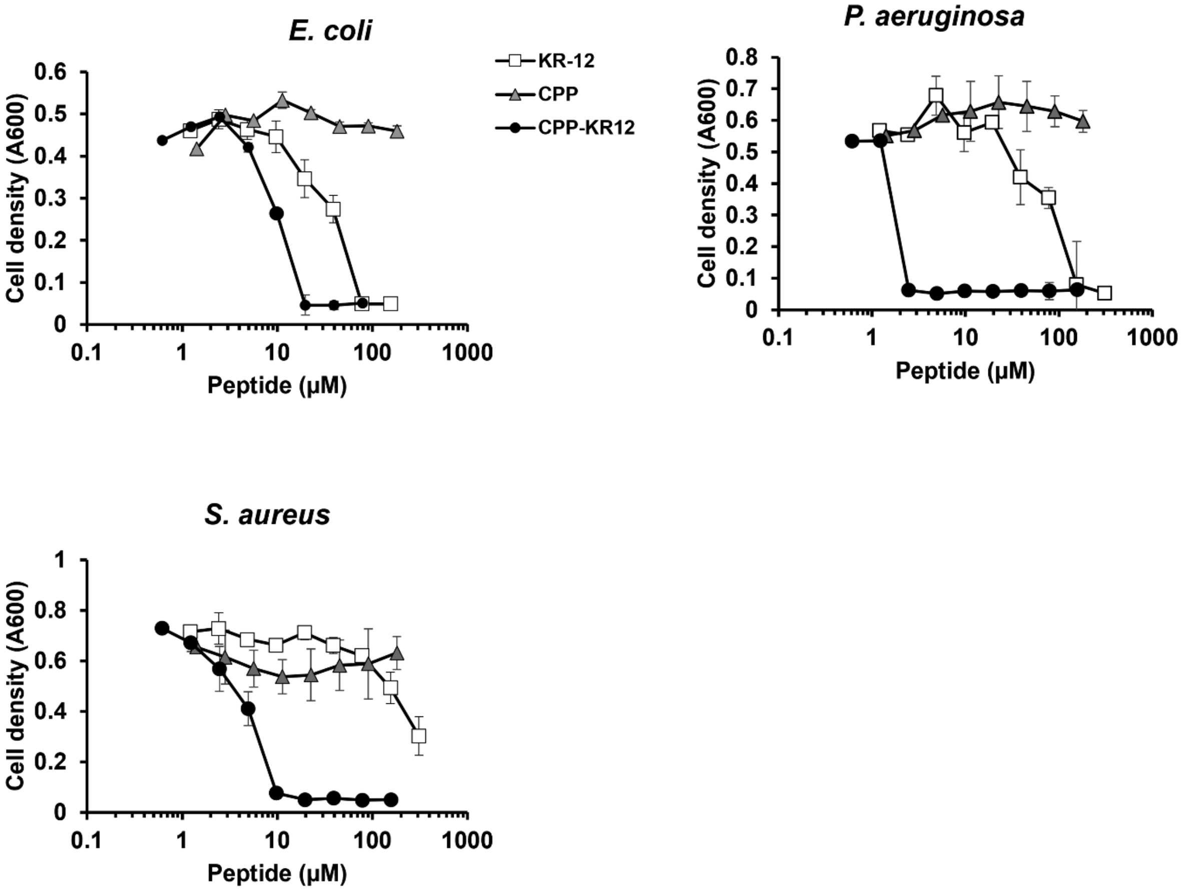

Figure S1. Antibacterial activity of indicated peptide depending on concentration

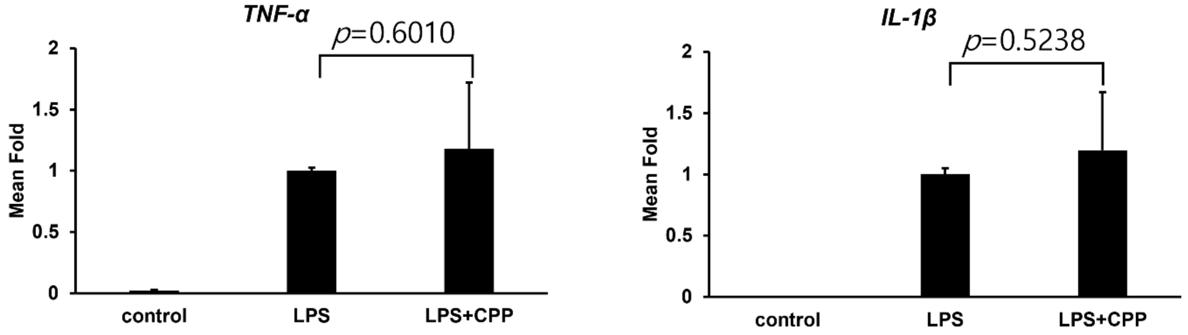

Figure S2. Anti-inflammatory response of CPP peptide in LPS-stimulated RAW264.7 cells. The mRNA expression levels of inflammatory cytokines, namely TNF-α and IL-1β, were measured by qRT-PCR. The Glyceraldehyde-3-Phosphate-Dehydrogenase (GAPDH) gene was used for normalization in gene expression. The gene levels in each treated cell were calculated relative to those in cells exposed to LPS without CPP. Values are presented as the mean ± SE (N = 3).

Table S2. Probability table for determining the significance of the difference in average minimum inhibitory concentrations across antimicrobial peptides for each strain.

		Bonferroni		
		p value		
		KR12	CPP-KR12	KR12@Si
E. coli	KR12			
	CPP-KR12	0.0447		
	KR12@Si	1	0.0401	
	CPP-KR12@Si	0.0514	1	0.0461
P. aeruginosa	KR12			
	CPP-KR12	0.0396		
	KR12@Si	1	0.0420	
	CPP-KR12@Si	0.0424	1	0.0449
S. aureus	KR12			
	CPP-KR12	0.0007		
	KR12@Si	0.0013	1	
	CPP-KR12@Si	0.0008	1	1

For multiple comparison, Two-Way Analysis of Variance (ANOVA), coupled with Bonferroni post hoc test was performed. Statistical significance was set at $p < 0.05$.

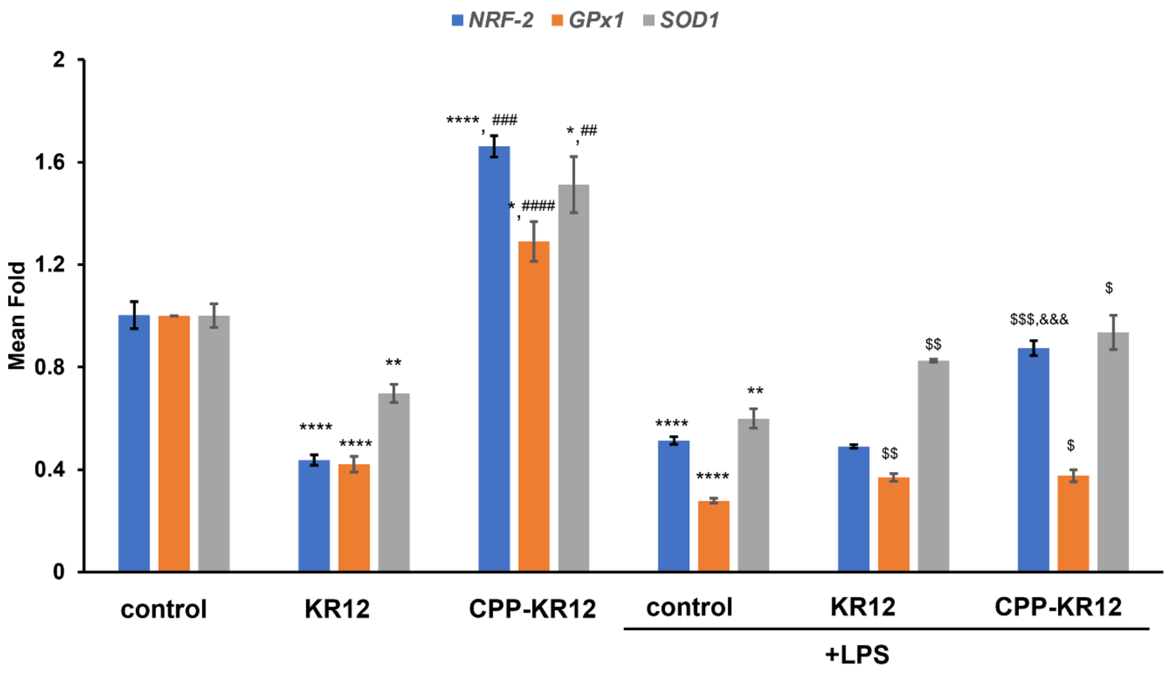

Figure S3. Anti-oxidant protein expression in response of AMPs in the presence or absence of lipopolysaccharide (LPS) in RAW264.7 cells. The mRNA expression levels of nuclear factor erythroid 2-related factor 2 (NRF-2), glutathione peroxidase 1 (GPx-1) and superoxide dismutase 1 (SOD1) were measured by qRT-PCR. Glyceraldehyde-3-Phosphate-Dehydrogenase (GAPDH) gene was used for normalization in gene expression. Gene levels in each treated cell were calculated relative to those in cells without AMP. Values are presented as mean ± SE (N = 3).$^{*}p<0.05$, $^{**}p<0.01$ and $^{****}p<0.0001$ vs control and $^{\#\#}$ $p<0.01$, $^{\#\#\#}p<0.001$, and $^{\#\#\#\#}p<0.0001$ vs KR12. $^{\$}p<0.05$, $^{\$\$}p<0.01$ and $^{\$\$\$}p<0.001$ vs control +LPS and $^{\&\&\&}p<0.001$ vs KR12+LPS Used primers as follows; NRF-2 (forward: ATGGACTTGGAGTTGCCACC; reverse: CCTGTTCCTTCTGGAGTTGCT), GPx1 (forward: CTGAATTCCCTCAAGTACGTCC; reverse: GCCCACCAGGAACTTCTCAAAG), and SOD1 (forward: GTGCGTGCTGAA GGGCGA; reverse: CCACCTTTGCCCAAGTCATC) Glyceraldehyde-3-phosphate dehydrogenase (GAPDH) (forward: CCTGGCCAAGGTCATCCATG; reverse: GCAGGAGACAACCTGGTCCT).

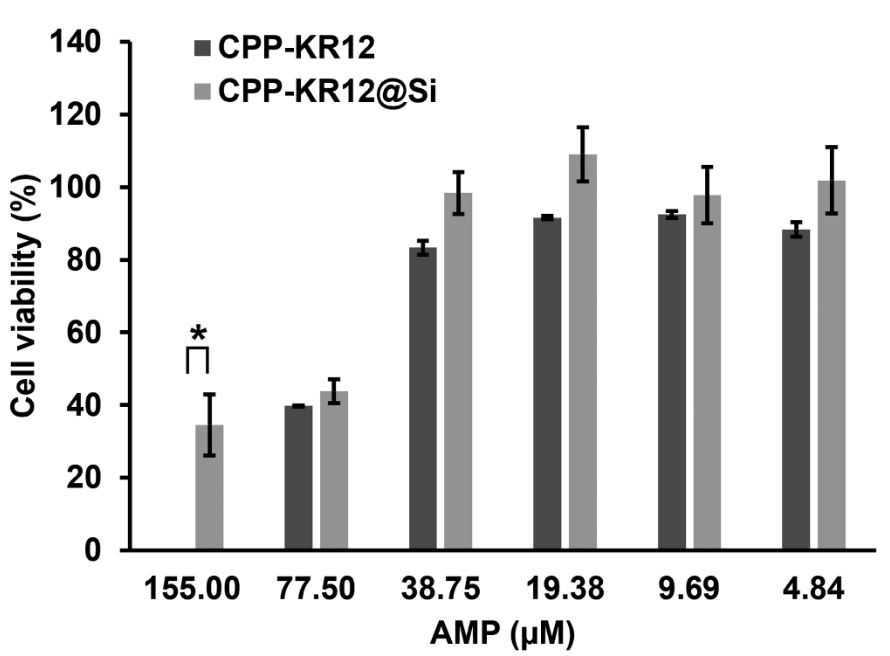

Figure S4. Comparison of cell viability after 5 days of AMP treatment. The cytotoxicity of AMP was measured 5 days after incubation of Raw 264.7 cells (4×10^5 cells/ mL) in the presence of each indicated concentration of AMP Cell survival was expressed as a percentage of the negative control grown without AMP. Values are presented as mean ± SE (N = 3). *$p < 0.05$ vs corresponding free form of AMP in each concentration.
